# Comparison of external system and implanted system in intrathecal therapy for refractory cancer pain in China: A retrospective study

**DOI:** 10.1002/brb3.2851

**Published:** 2022-12-21

**Authors:** Wangjun Qin, Li Zhao, Botao Liu, Yang Yang, Peng Mao, Liyuan Xu, Pengmei Li, Yongguang Shang, Lei Zhang, Bifa Fan

**Affiliations:** ^1^ Department of Pharmacy China‐Japan Friendship Hospital Beijing China; ^2^ Department of Pain Management China‐Japan Friendship Hospital Beijing China

**Keywords:** comparative analysis, intrathecal therapy, refractory cancer pain, risk management

## Abstract

**Introduction:**

Intrathecal therapy (ITT) via an implanted system was demonstrated for the treatment of refractory cancer pain for decades. Recently, the dissemination of ITT is enhanced in an external system way in Asia for a lower implantation cost. This study compares the efficacy, safety, and cost of the two ITT systems in refractory cancer pain patients in China.

**Methods:**

One hundred and thirty‐nine cancer pain patients who underwent implantation of the ITT system were included. One hundred and three patients received ITT via the external system (external group), while 36 patients received ITT via the implanted system (implanted group). A 1:2 propensity score matching procedure was used to yield a total of 89 patients for the final analysis. Medical records of included patients were retrospectively reviewed and pain scores, incidences of complications, and costs were compared.

**Results:**

ITT via the external system provided pain relief as potent as ITT via the implanted system but was less time‐consuming in the implantation phase (13 vs. 19 days, *p* < .01). Nausea/vomiting and urinary retention were the most frequent adverse events in both external and implanted groups (32.14%, 16.07% vs. 36.36%, 21.21%). No significant difference was found in the incidences of all kinds of complications. Compared to the implanted group, the external group cost less for the initial implantation (7268 vs. 26,275 US dollar [USD], *p* < .001) but had a significant higher maintenance cost (606.62 vs. 20.23 USD calculated monthly, *p* < .001).

**Conclusions:**

ITT via the external system is as effective and safe as that via the implanted system and has the advantage of being cheap in the upfront implantation but costs more during the maintenance process in China.

## Graphical abstract

Intrathecal therapy (ITT) via an implanted system was demonstrated for the treatment of refractory cancer pain since decades. ITT via the external system is as effective and safe as that via the implanted system and has the advantage of being cheap in the upfront implantation, but cost more during the maintenance process in China.ITT via the external system could be an alternative approach for refractory cancer pain patients in China.

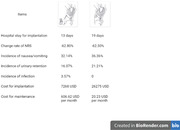

## 1 INTRODUCTION

Pain occurs in up to 70% of cancer patients and seriously affects the patients’ mood and quality of life (Haumann et al., 2017). Although the World Health Organization cancer pain ladder is extensively employed, it remains 10%–20% of cancer pain patients being inefficiently treated (Afsharimani et al., 2015). Intrathecal therapy (ITT), which directly delivers opioids such as morphine into the cerebrospinal fluid (CSF), has demonstrated its effectiveness on refractory cancer pain (Bhatia et al., 2013; De Andres et al., 2020; Deer et al., 2017; Sayed et al., 2018).

ITT for cancer pain was first reported in 1977, and the first case of ITT via an implanted pump was published in 1981 (Onofrio et al., 1981). Since then, ITT has been seen as the “last resort” option for refractory cancer pain (De Andres et al., 2020), which has a poor response to systemic opioid therapy (Smith et al., 2002). ITT via an external system infuses opioids into CSF through a percutaneous port connecting to the end of intrathecal catheter (De Andres et al., 2020) and is now widely used in Asia for its lower implantation cost (Hattori et al., 2009; J. H. Kim et al., 2013; Zheng et al., 2017). However, concerns still exist about the efficacy, safety, and cost utilization regarding ITT via the external system.

In terms of efficacy, as the external pump delivers opioid diluted in 0.9% sodium chloride solution in a larger volume, intrathecal opioid may less localize to the target receptors of the spinal segments associated with the dermatomal area of pain in the external system for the positive relation between the infusion volumes and the spread of intrathecal infusion in the intrathecal space (Dupoiron, 2019; Tangen et al., 2017). Also, the risks of complications such as infection and CSF leak are supposed to be higher in ITT via the external system (Bhatia et al., 2013; De Andres et al., 2020), which will further affect the choices of patients and clinicians. Moreover, although the external system has a lower initial implantation cost, its maintenance cost is robustly higher than that of the implanted system in developed countries such as the United States (Bruel & Burton, 2016; De Andres et al., 2020). However, there is scarce knowledge about the implantation and maintenance costs for the two ITT systems in China.

In this study, we conducted a direct comparison between the two ITT systems by retrospectively reviewing cancer pain patients who underwent ITT. The clinical efficacy and the incidences of complications, as well as the costs of ITT, were assessed and compared, which would provide a comprehensive and full‐scale understanding for patients and clinicians in China when justifying treatment decisions.

## 2 MATERIAL AND METHODS

### 2.1 Study setting and design

This retrospective analysis was conducted at the China‐Japan Friendship Hospital in China from the provider's perspective in accordance with the Declaration of the World Medical Association. The ethical committee of China‐Japan Friendship Hospital approved this study and waived the need for informed consent (Registration number: 2020‐103‐K67). Cancer pain patients who underwent implantation of ITT system via an external pump (external group) or an implanted pump (implanted group) at the hospital from November 2019 to November 2021 were included in the study. Patients not initiating the ITT during the hospitalization, as well as those with incomplete case data, were excluded from the study. This excluded four patients in the implanted group and 14 patients in the external group. Patients were then matched based on demographic characteristics using the propensity score (PS) matching technique to reduce bias. The nearest available matching was performed with matching ratio 1:2 and 0.2 caliper by controlling the following variables: body mass index (BMI), Karnofsky performance scores (KPS), and numerical pain rating scales (NRS) scores.

### 2.2 Implantation of ITT system

The implantation of ITT via an external pump included tunneling the distal end of the intrathecal catheter to the anterior wall of the abdomen and connecting it to a subcutaneous port (C. R. Bard, Inc.). An external drug infusion pump (Hospira Inc.) infuses opioids into intrathecal space through a butterfly‐winged needle vertically inserting into the subcutaneous port. The distal end of the intrathecal catheter of ITT via an implanted pump was connected to a programmable‐flow pump (Medtronic, Inc.) implanted subcutaneously in the side of the abdomen. The same antibiotics dosing regimen was applied to prevent post‐operative infection in both groups. After the successful implantation of the ITT system, intrathecal opioid was titrated according to guidelines and clinical experience, and systemic opioid was then stopped or quickly weaned off before hospital discharge.

### 2.3 Data collection

Medical records of included patients were retrospectively reviewed. Demographic information, operation data, and complications related to ITT were obtained from the medical records. NRS scores, KPS, and doses of opioids before and after ITT were determined at the time of admission and discharge, respectively. Medical records for the management of complications after ITT was also reviewed.

### 2.4 Cost calculation

The costs of ITT included the implantation cost and the maintenance cost. The implantation cost was determined as the hospitalization expense, including the cost of subcutaneous port or programmable‐flow pump, ward fee, image examination fee, anesthetic fee, operation fee, and so on. The maintenance cost included the registration fee, professional fee for refilling medication, refill kit fee, and cost of medication. The cost of medication was calculated based on the doses of intrathecal opioid at discharge for the first month. The costs of the registration fee, professional fee for refilling medication, and refill kit fee were averaged over a month according to the interval of visits, which was 15 days for the external group and depended on the doses of intrathecal opioid for the implanted group, with a maximum of 180 days.

### 2.5 Statistical analysis

Patient characteristics were expressed as median (25th quartile, 75th quartile) or number (%). The cost was calculated in US dollar (USD), using a reference of USD 1 = Chinese yuan 6.50 (Chinese reals). Continuous variables were compared by Mann–Whitney U‐test between the external and implanted groups, and by Wilcoxon matched‐pairs signed rank test between before and after ITT for each group. Categorical variables were analyzed using the chi‐square test or Fisher's exact test. Statistical analyses were conducted using SPSS version 22.0 (IBM Corp.). The significant level was taken as *p* < .05.

## 3 RESULTS

### 3.1 Patient demographics

A total of 139 cancer pain patients with ITT were included in the present study, 103 in the external group and 36 in the implanted group. There were no statistical differences between the external and implanted groups in terms of age, gender, pain duration before ITT, oral morphine equivalence dose, reason for ITT, or type of cancer. However, lower BMI and KPS score and higher NRS score were observed in the external group as compared to the implanted group (*p* = .0139, .0211, and .0050, respectively). After the PS matching process, 33 patients in the implanted group were successfully matched to 56 patients in the external group, and all demographic characteristics were well balanced between the matched groups (Table 1).

**TABLE 1:** Demographic characteristics of patients with ITT via the external system or the implanted system

	Before propensity score (PS) matching			After PS matching		
Parameter	Implanted group *n* = 36	External group *n* = 103	*p*‐value	Implanted group *n* = 33	External group *n* = 56	*p*‐value
Age, year	59 (55, 66)	62 (54, 67)	.6285	59 (55, 66)	62 (52, 65)	.9983
Gender (M/F)	21/15	57/46	.7554	21/12	27/29	.1586
BMI, kg/m^2^	22.48 (20.76, 23.92)	20.51 (17.93, 20.52)	.0139	22.47 (20.77, 23.51)	21.32 (18.35, 23.05)	.1073
KPS score	90 (70, 100)	75 (55, 95)	.0211	90 (70, 100)	90 (70, 100)	.8874
NRS score	6 (5, 7)	7 (6, 7)	.0050	6 (6, 7)	7 (5, 7)	.7050
Pain duration before ITT, year	0.75 (0.29, 1.88)	0.5 (0.33, 1)	.1212	1.00 (0.33, 2.00)	0.50 (0.33, 1)	.1608
OMED, mg/day	225 (80, 660)	200 (80, 480)	.8868	240 (80, 660)	175 (80, 600)	.9612
Reason for ITT, %						
Inadequate pain relief	31 (86.11)	76 (73.79)	.1305	29 (87.88)	44 (78.57)	.2694
Intolerance of drug‐related toxicity	5 (13.89)	27 (26.21)		4 (12.12)	12 (21.43)	
Type of cancer, %						
Digestive system	17 (47.22)	33 (32.04)	.1111	15 (45.45)	18 (32.14)	.2581
Respiratory system	13 (36.11)	30 (29.13)	.5302	12 (36.36)	19 (33.93)	.8220
Reproductive system	2 (5.56)	17 (16.50)	.1568	2 (6.06)	7 (12.50)	.4754
Hepatobiliary system	1 (2.78)	9 (8.74)	.4532	1 (3.03)	7 (12.5)	.2494
Urinary system	1 (2.78)	7 (6.80)	.6800	1 (3.03)	2 (3.57)	.9999
Other	2 (5.55)	7 (6.80)	.9999	2 (6.06)	3 (5.36)	.9999

Abbreviations: BMI, body mass index; ITT, intrathecal therapy; KPS, Karnofsky performance scores; M/F, male/female; NRS, numerical pain rating scales; OMED, oral morphine equivalence dose.

### 3.2 Implantation parameters of the matched sample

**FIGURE 1:**
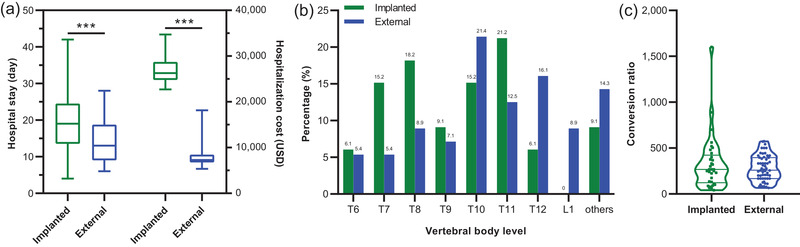
Comparison of implantation parameters between the external and implanted groups. Significant difference was found in hospital stay and hospitalization cost (a), but not in the location of intrathecal catheter tips (b), and conversion ratios of doses from systemic to intrathecal opioids (c). ***p < .001 versus the implanted group. USD, US dollar

ITT via the external system required a shorter hospital stay (13 vs. 19 days, *p* < .01) and a lower hospitalization cost (7268 vs. 26,275 USD, *p* < .001) for implantation than ITT via an implanted pump (Figure 1a). The location of intrathecal catheter tips did not differ significantly between the external and implanted groups, most of which were located at the levels from T6 to L1 vertebral body (Figure 1b). Also, no significant difference was observed in the conversion ratios of doses from systemic to intrathecal opioids between the external and implanted groups (Figure 1c).

### 3.3 Efficacy of ITT in the matched sample

**FIGURE 2:**
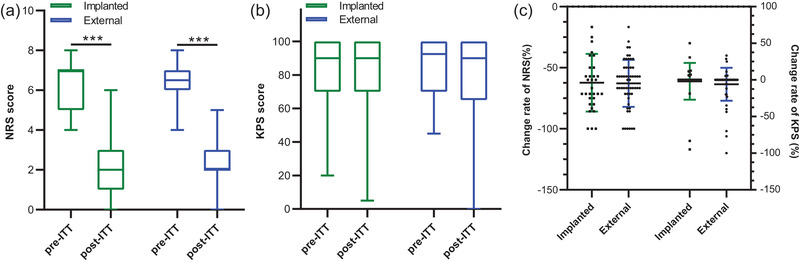
Comparison of efficacy of ITT between the external and implanted groups. Both groups exhibited significant pain relief after ITT (a), while a trend of decrease in KPS score after ITT was only observed in the external group (b). No significant difference was found in change rates of both NRS and KPS scores between two groups (c). *p < .05; ***p < .001 versus pre‐ITT. ITT, intrathecal therapy; NRS, numerical pain rating scales; KPS, Karnofsky performance scores

Both the external and implanted groups showed a robust decrease in the NRS score (external: 6.5 vs. 2, *p* < .001; implanted: 7 vs. 2, *p* < .001) after ITT as compared to that before ITT (Figure 2a). In terms of KPS score, a trend of impairment in the activity of daily living was observed in the external group but failed to reach conventional levels of statistical significance (92.5 vs. 90, *p* = .085, Figure 2b). There is no significant difference either in the change rate of KPS score or in the change rate of NRS score between the external and implanted groups (Figure 2c).

### 3.4 Complications of ITT in the matched sample

**FIGURE 3:**
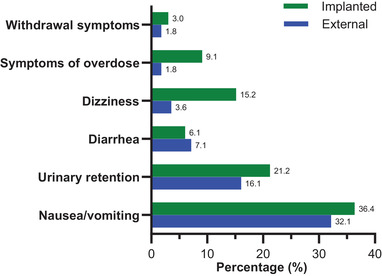
Comparison of incidences of intrathecal opioid‐induced adverse events between external and implanted groups. Nausea/vomiting and urinary retention were the most frequent intrathecal opioid‐induced adverse events in both groups. There is no significant difference in the incidences of adverse events between the external and implanted groups

The main complications of ITT included intrathecal opioid‐induced adverse events and surgical complications. Nausea/vomiting and urinary retention were the most frequent intrathecal opioid‐induced adverse events in both external (18, 32.14% and nine, 16.07%, respectively) and implanted groups (12, 36.36% and seven, 21.21%, respectively). Other intrathecal opioid‐induced adverse events included diarrhea, dizziness, pruritus, symptoms of overdose including somnolence and dyspnea, and the withdrawal symptoms including anxiety, restlessness, and palpitations (Figure 3). All the adverse events were subsided with supportive care or adjusting the dosage of intrathecal opioids. Post‐dural puncture headache possibly due to CSF leak was observed in one (1.78%) patient in the external group and one (3.03%) patient in the implanted group, all of which were managed conservatively with bed rest and increased fluid intake. Wound discharge around the pump pocket occurred in one patient in the implanted group 3 months after ITT, which was managed by debridement and suture. Two patients in the external group suffered from surgical site infection after ITT. One patient infected 2 weeks after ITT was treated with antibiotics, while the other who became infected 4 weeks after ITT required the removal of the implanted port and intrathecal catheter to clear up the infection. There was no significant difference in the incidence of serious adverse events including post‐dural puncture headache, wound discharge, and surgical site infection.

### 3.5 Cost analysis in the matched sample

The cost of ITT includes the implantation cost and the maintenance cost. The implantation cost in the external group was significantly lower than that in the implanted group (7268 vs. 26,275 USD, *p* < .001, Figure 1a). The maintenance costs included the pump rental, ITT opioid cost, and the refill cost (Table 2). Patients with the external ITT system needed to rent an external pump at a cost of 484.62 USD per month. The refill cost in the external and implanted groups was 119.29 (119.29, 119.29) USD and 16.67 (16.67, 20.24) USD per month on average, respectively (*p* < .001; Table 2). ITT opioid cost per month was 2.35 (1.17, 4.69) USD in the external group and 3.65 (3.65, 4.78) USD in the implanted group (*p* = .4906). Overall, the maintenance costs of the external group were significantly higher than those of the implanted group (606.62 vs. 20.23 USD calculated monthly, *p* < .001).

**TABLE 2:** Maintenance cost of ITT via the external system or the implanted system per month on average

Maintenance cost (USD)	Implanted group *n* = 33	External group *n* = 56
Pump rental per month	0 (0, 0)	484.62(484.62, 484.62)
ITT opioid cost per month	3.65 (3.65, 4.88)	2.71 (2.71, 6.23)
Refill cost calculated for a month	16.67 (16.67, 20.24)	119.29 (119.29, 119.29)
Registration fee for a refill	7.69 (7.69, 7.69)	7.69 (7.69, 7.69)
Professional fee for refilling medication	46.15 (46.15, 46.15)	3.69 (3.69, 3.69)
Refill kit	46.15 (46.15, 46.15)	49.03 (49.03, 49.03)
Refill period (day)	180 (160, 180)	15 (15, 15)

Abbreviations: ITT, intrathecal therapy USD, US dollar.

## 4 DISCUSSION

ITT has been well established as an effective tool for the treatment of refractory cancer pain, but the high cost for implantation of the implanted system often impeded its implementation (Deer et al., 2017; Ontario, 2016). The present study shows that ITT via the external system could be an alternative approach for patients with cancer pain as it required a lower initial implantation cost but provides the same degree of pain relief without causing a higher risk of complication. To the best of our knowledge, the present study is the first comprehensive comparison between ITT via the external system and ITT via the implanted system in patients with cancer pain in China, which could be helpful in making a clinical decision in clinical practice.

The biggest advantage of ITT is the superior analgesic effect produced by smaller doses of opioids (De Andres et al., 2020). The effectiveness of ITT is to some degree associated with the congruence between catheter placement and dermatomal area of pain, as there is little bulk flow in CSF and significant concentration gradients from the catheter tip (De Andres et al., 2020; Deer et al., 2017; Kroin et al., 1993). The congruence between catheter placement and the dermatomal area of pain is also conducive to the precise targeted delivery of lipophilic medications such as local anesthetics (De Andres et al., 2020). In this study, positions of catheter tips were mostly located between T6 and L1 in both the external and implanted groups, mainly depending on the pain‐producing area. It is reported that the spread of intrathecal infusion is positively associated with the infusion volumes in the reconstructed human central nervous system model (Dupoiron, 2019; Tangen et al., 2017). Therefore, intrathecal opioid may less localize to the site of infusion in the external group as the external pump has to deliver opioid diluted in 0.9% sodium chloride solution in a larger volume for a fewer precision. However, the present study shows no difference in the effectiveness between the external and implanted groups when evaluated with either the decreased rate of NRS score or the conversion ratio between doses of systemic and intrathecal opioids (Gorlin et al., 2016). The disadvantage of ITT via an external pump is that patients must wear an external drug infusion pump and receive meticulous injection site care such as daily injection site disinfection, change of the butterfly‐winged needle in each visit, and avoiding inadvertent catheter dislodgement or removal in daily life, which could impair the performance of daily activity and further influence life satisfaction and affective experience (Bhatia et al., 2013; De Andres et al., 2020). The present study shows that ITT via the external system had a trend to decrease patients’ KPS scores, which is in accordance with a previous study and further proves the disadvantage of ITT via the external system (Qin et al., 2020).

The complications related to ITT could be more of a concern when considering ITT via the external system (Bhatia et al., 2013). The risk of infection is a key concern. In the present study, the incidence of infection is very low in the external group and shows no significant difference as compared to the implanted group. This profits partly from the utilization of the subcutaneous port that avoids a direct connection between the external infusion system and the intrathecal space (De Andres et al., 2020). Other reasons include the regular disinfection measures and change of the external infusion tube in every visit with an interval of 2 weeks, care transitions in the long‐term ITT, and the careful prevention measures in daily life (Qin et al., 2020). The most frequent surgical complication was post‐dural puncture headache in both groups, which suggests no significant difference in the risk of pericatheter CSF leakage between the two ITT systems (Adler & Lotz, 2017; Peralta et al., 2020). The incidences of intrathecal opioid‐induced adverse events are much the same in the two groups, and the most frequent adverse events were nausea/vomiting and urinary retention, which is in accordance with other studies (J. H. Kim et al., 2013; Mastenbroek et al., 2017; Qin et al., 2020). Thus, frequent monitoring and active treatment of nausea/vomiting and urinary retention are very important for improving patients’ compliance and acceptance at the beginning of ITT, in which the pain management interdisciplinary team including pharmacists could make contributions in many respects.

The cost burden of ITT is also an important impediment to the implementation of ITT, which includes a high implantation cost and a low maintenance cost (Kleinmann & Wolter, 2017). Despite the low maintenance cost, ITT via the implanted system is indeed a costly therapy. However, little has been reported about the cost‐effectiveness of the implanted system, compared with conventional therapy (Duarte et al., 2018; Ontario, 2016). Cost benefit is predicted at 34.2 months in the United States, 28 months in Canada, and 24.2 months in Korea (Brogan et al., 2013; E. J. Kim et al., 2016; Kumar et al., 2002). Our previous study demonstrated that a median interval of 11.44 months is needed for ITT via the external system to be cost‐effective, compared with conventional therapy (Qin et al., 2020), which is in accordance with the lower implantation cost of external ITT system in the present study. However, ITT via the external system needs a significantly higher maintenance cost in China, which may affect the establishment of cost‐effectiveness, compared with ITT via the implanted system. When compared with epidural morphine therapy via an external pump, ITT via the implanted system would be more cost‐effective after an interval time of 3–6 months in cancer pain patients (Bedder et al., 1991; Mueller‐Schwefe et al., 1999). Considering the high maintenance cost and opioid doses in epidural morphine therapy via an external pump (D. D. Kim et al., 2019), these results cannot tell us which one is more cost‐effective between ITT via the external system and ITT via the implanted system. Thus, a direct comparison of cost‐effectiveness between the two ITT systems should be further studied.

There are still several limitations in the present study. First, as it is a retrospective study, the two groups were not matched with regard to some baseline characteristics such as the number of patients, BMI, and NRS score. The differences in characteristics between the two groups, on the one hand, may have an influence on the outcomes, and on the other hand, reflect the important considerations of patients when selecting the procedure of ITT. Cancer pain patients in good shape physically may tend to choose ITT via an implanted pump for greater mobility. In this study, we addressed this limitation by employing a 1:2 PS matching procedure. Second, the results in the present study were based only on Chinese data. Therefore, the applicability of cost comparisons in other countries is limited. In this regard, similar research should be conducted in a wider range of countries. Finally, we cannot analyze the long‐term cost‐effectiveness, as most of the patients passed away in a median of 5.7 months. According to the results of the present study, ITT via the external system seems a cost‐saving choice for patients with limited life expectancy as the difference of the implantation cost is far larger than that of maintenance cost.

## 5 CONCLUSION

In summary, the present study provides the first evidence on the comprehensive and realistic comparison of efficacy, safety, and cost between ITT via the external system and the implanted system. ITT via the external system balances the high cost of ITT via the implanted system with the poor pain relief of systemic opioid therapy for refractory cancer pain, without increasing the incidences of complications, which could be an alternative approach for refractory cancer pain patients in China.

## CONFLICT OF INTEREST

No potential conflict of interest was reported by the authors.

### PEER REVIEW

The peer review history for this article is available at: https://publons.com/publon/10.1002/brb3.2851

## Data Availability

Dataset might be available to interested researchers on request from the authors.
